# Does patient and public involvement impact public health decision-making? A 10 year retrospective analysis of public consultation in Brazil

**DOI:** 10.1186/s12961-023-01018-1

**Published:** 2023-07-12

**Authors:** Ana Carolina De Freitas Lopes, Hillegonda Maria Dutilh Novaes, Patrícia Coelho De Soárez

**Affiliations:** 1 Departamento de Medicina Preventiva, Faculdade de Medicina da Universidade de São Paulo, Av. Dr. Arnaldo 455, Cerqueira Cesar, São Paulo, SP 01246903 Brazil; 2 Departamento de Gestão e Incorporação de Tecnologias em Saúde, Ministry of Health, Esplanada dos Ministérios, Bloco G, Brasilia, Brazil

**Keywords:** Patient and public involvement, Stakeholder involvement, Public consultation, Decision-making, Health technology assessment, Logistic regression

## Abstract

**Background:**

The aim of this work is to characterize the processes associated with patient and public involvement (PPI) in the form of public consultations (PC) during the first 10 years of operation of the National Committee for Health Technology Incorporation in the Unified Health System (Conitec) of Brazil, and to identify factors associated with changes in Conitec’s recommendations following these PC.

**Methods:**

This cross-sectional study analysed all processes related to the adoption of technologies that took place in Brazil between 2012 and 2021 based on technical reports and self-reported information collected from PC participants. A multiple logistic regression model identified factors associated with changes in Conitec’s recommendations following PC.

**Results:**

A total of 479 technical reports were published, of which 83% (*n* = 400) were submitted to PC. Demands were made mainly by applicants from the government (*n* = 262; 55%), regarding the adoption of medicines (*n* = 366; 76%), in which context neoplasms and infectious diseases were the most frequent indications (*n* = 66; 14% for each). A total of 264 (55%) processes resulted in a final recommendation in favour of introducing the technology. Over the period of 10 years, 196 483 contributions were received in response to PC. The largest volume of contributions was made by patients and their families or representatives (*n* = 99 082; 50%), females (122 895; 67%), white individuals (129 165; 71%) and individuals between the ages of 25 and 59 years (145 364; 80%). Alteration of the preliminary recommendation occurred in 13% (*n* = 53) of the PC, with a higher proportion of recommendations being altered from 2017 onwards. Increased participation by patients had a significant impact on the alteration of the preliminary recommendation (odds ratio 3.87, 95% CI 1.33–13.35, *p* = 0.02).

**Conclusions:**

Increased engagement of patients and their families and caregivers in PC was associated with changing the preliminary recommendation of Conitec about the adoption of technologies into the public health system in Brazil.

**Supplementary Information:**

The online version contains supplementary material available at 10.1186/s12961-023-01018-1.

## Background

The current health system of Brazil was proposed as part of the country’s process of democratization and based on a movement led by civil society and public health professionals. The 1988 Brazilian Constitution established the Unified Health System (SUS) and mandated that health is a fundamental right of all citizens and a responsibility of the state. The principles of SUS include universality and comprehensiveness of care, which are guided by a decentralized and participatory management system [1]. Although the Brazilian health system is universal, it comprises a combination of public and private organizations. Within the public health sector, all individuals have free and open access to health services and products. The financing of SUS is a shared responsibility of the union, states and municipalities. The participatory component of SUS is mainly based on health conferences and health councils at the three level of government, as mandated by law since 1990, which enhanced the diversity and impact of stakeholders’ engagement in decision-making [1].

The adoption of technologies into the SUS must observe scientific criteria based on health technology assessment (HTA), including aspects related to efficacy, effectiveness, accuracy and safety as well as efficiency (cost-effectiveness analysis) and affordability (budget impact analysis) [2]. Those criteria are similar to the ones observed in the UK, Canada and Australia [3]. The assessment of health technologies to determine whether they should be offered by the public health sector, from primary care guidelines to gene therapies, is conducted by the National Committee for Health Technology Incorporation in the Unified Health System (Conitec) [3].

Conitec is an advisory body associated with the Brazilian Ministry of Health (MoH), which is composed of three committees with 15 members each. Conitec’s members represent the MoH (seven members), public health managers (two members), regulatory agencies of the sector in the country (two members), the Federal Council of Medicine (CFM) (one member), the Brazilian Medical Association (AMB) (one member), institutions from the Brazilian Network for the Evaluation of Health Technology (REBRATS) (one member) and the national health council (one member), which includes other health professionals, SUS users and the private sector [4].

Members of Conitec are appointed by the heads of each representative institution and must have professional experience or academic training in HTA. Membership is unpaid and driven by public interest. To ensure transparency, all members must disclose their conflicts of interest with each recommendation issued by Conitec. The MoH funds the activities necessary to carry out Conitec’s work [4].

The process of adopting of a health technology into the SUS begins when a stakeholder formally requests it, providing clinical and economic evidence to support the claim. Technical professionals from the MoH or REBRATS, chosen for their expertise, analyse the demand and issue a report for deliberation by Conitec members. After appraising and discussing the report’s findings, Conitec issues a preliminary recommendation for or against the adoption of the technology into the SUS.

The preliminary recommendation and the studies used to support it are then opened up for an online public consultation (PC) where anyone can contribute. Technical professionals responsible for the initial report analyze and summarize the contributions from PC. While there are no official guidelines on how to analyse the contributions from PC, Conitec uses them to make its final recommendation.

After examining the contributions elicited by PC, Conitec issues its final recommendation, which can either maintain or change its preliminary recommendation, that is, in favour or against the adoption of the technology. If needed, a public hearing may be conducted before the MoH makes the final decision. Public and patient involvement (PPI) is, therefore, a unidirectional flow of information, from society to Conitec.

**Fig. 1:**
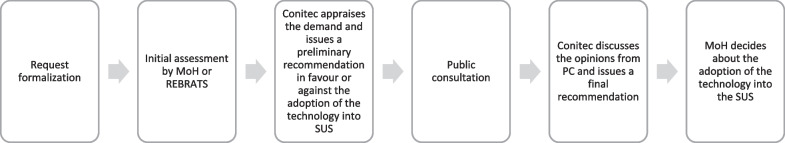
Flowchart of the steps involved in the process of adopting technologies into the SUS

All demands that meet the formal requirements must be analyzed and decided within 270 days, with no prioritization criteria beyond meeting deadlines. Figure 1 provides a flowchart of the steps involved in the process, from the initial demand to final decision on adopting technologies into the SUS. Studies analysing patient and public involvement with Conitec, including observations collected up to 2018, have identified that PPI features people who play the roles of incorporation demanders, participants as fixed members in the deliberative plenary, participants in consultations and participants in public hearings [5, 6]. The impact of PPI implemented by Conitec was measured by reference to the perceptions of stakeholders and the number of contributions received in response to public consultations [5, 7]. Silva et al. noted that the stakeholders involved in this process viewed Conitec as promoting participation in an important way, even providing opportunities for improvement [7].

PPI implemented by Conitec is aligned with the growing movement to encourage greater inclusion of society in health decisions [8, 9]. The aim of this movement is to improve the accountability and legitimacy of the actions taken as well as to ensure the quality, efficiency and adequacy of the results of the decisions made [8, 10]. However, the impact of PPI on health decisions, which has both a complex and a contextual nature, is not yet well understood or measured [11–13].

The aim of this work is to characterize the process underlying PPI in terms of public consultations during the first 10 years of Conitec’s operations and to identify factors associated with alterations in Conitec’s preliminary recommendations regarding the adoption of technologies into the SUS, in particular those related to the influence of different stakeholders.

## Methods

This study features a cross-sectional design and adopts a quantitative approach to investigate the processes associated with the adoption of technologies into the SUS. A retrospective analysis was conducted to investigate all processes associated with the adoption of technologies into the SUS for which a final decision was published between January 2012 and December 2021, a period corresponding to the first 10 years of Conitec’s operations. Technical reports on the corresponding medications, products or procedures that were available on the institution’s website (https://www.gov.br/conitec/pt-br) or from anonymized databases containing the contributions sent in response to public consultations conducted during the period were used as data sources.

Data extraction for each demand analysed by Conitec was performed using a specific data extraction worksheet in Microsoft Excel®, which contained information regarding the analysed technology, the proposed indication, the incorporation demander, Conitec’s preliminary and final recommendations, the decision of the MoH, the opening and closing dates of the process, the total number of contributions in response to the public consultation and the self-declared characteristics of the participants who made these contributions (see Additional file 1).

The self-declared characteristics of the participants were the interest group with which they identified as well as their gender, colour, age and state of residence. To preserve the anonymity of the participants, this information was already categorized in the publicly accessible version, as presented in the Results section. Data were extracted by the main researcher and two research assistants. The consistency of the data extraction process was verified in the final database by the main researcher.

For data analysis, technologies were categorized as medicines, procedures or products. The indications of the requests for incorporation were grouped based on the chapters of the International Classification of Diseases (tenth revision). The demanders referred to the proponents of the applications for the adoption of these technologies. For this analysis, demanders were classified into four categories according to their origin: government, private sector, social or mixed. Demanders with a government origin corresponded to public institutions, such as public health managers, judiciary and public health institutions. Private sector demanders represented private institutions, including the pharmaceutical industry and private health institutions. Demanders with a social origin included civil society and organizations that were non-governmental actors and not members of the private sector, that is, patient associations, medical societies or isolated individuals. The category of mixed-origin demanders referred to any combination of demanders that were classified into different categories; for example, a claim filed by a company (private sector demander) in conjunction with a healthcare professional (social origin demander) would be classified as a mixed-origin demander.

Conitec’s recommendations were classified into two categories: changed or maintained. Recommendations were considered to have been changed if the final recommendation differed from the preliminary recommendation, for example, cases in which the preliminary recommendation was against the adoption of the technology and after the public consultation the final recommendation was in favour of the adoption of the technology. Recommendations in which the preliminary and final recommendations were the same were considered to have been maintained.

To identify the factors associated with the outcome ‘change in Conitec recommendation’ (binary outcome: yes – ‘changed recommendation’ and no – ‘maintained recommendation’), a crude analysis was conducted to investigate the relationships between each of the exposures and the outcome. Subsequently, a multiple logistic regression was conducted that included all exposure variables: type of technology, demander, indication and the volume of contributions by stakeholders in public consultations. Each technology incorporation process represented a unit of analysis.

Since the number of contributions per stakeholder in the same public consultation exhibited high variability, it was decided to create two groups, one of which made a large volume of contributions and one of which made a smaller volume of contributions. The cut-off point between these two groups was 100 contributions (the median number of contributions per public consultation was 80 (interquartile range 18–389), as shown in the Results section). Thus, a public consultation that received 150 patient contributions was classified as a public consultation with a large volume of patient contributions. If the same public consultation had received 20 contributions from institutions or health professionals, it would have been classified as a public consultation with a smaller volume of contributions from institutions or health professionals. That is, each public consultation was classified in terms of the volume of contributions made by each stakeholder independently. The purpose of this classification was to determine whether it would be possible to predict whether a recommendation would change based on the participation of a larger or smaller number of specific stakeholders.

## Results

During the first 10 years of Conitec’s operations, 479 reports containing analyses pertaining to the adoption of technologies into the SUS were published. In all cases, the MoH’s decision was in agreement with Conitec’s final recommendation. The median time taken by these analyses was 247 days (IQR 158–314 days), and 58% (*n* = 276) of the cases were analysed within the maximum period of 270 days stipulated by law.

Table 1 presents the characteristics of the technology incorporation processes based on the type of technology, demander, indication and final decision in question. Most of the analyses pertained to the incorporation of medicines (*n* = 366; 76%) and requests from the government, such as the MoH itself or local health management institutions (*n* = 262; 55%). Demands made by social representatives represented only 4% (*n* = 17) of the total. Neoplasms and infectious diseases were the indications associated with the highest frequency of demands, with 66 processes analysed (14%) for each. A total of 264 (55%) analyses made a final recommendation in favour of adopting the technology into the SUS.

**Table 1:** Characteristics of the technology incorporation processes into the SUS between 2012 and 2021

Characteristics	*N* (%)
*Type of technology*	
Medicines	366 (76)
Procedures	80 (17)
Products	33 (7)
*Demander*	
Government	262 (55)
Private sector	184 (38)
Social	17 (4)
Mixed	16 (3)
*Indication*	
Neoplasms	66 (14)
Infectious and parasitic diseases	66 (14)
Endocrine, nutritional and metabolic diseases	59 (13)
Diseases of the musculoskeletal system and connective tissue	52 (11)
Diseases of the nervous system	39 (8)
Diseases of the circulatory system	26 (6)
Diseases of the respiratory system	25 (5)
Others	136 (29)
*Final decision in question*	
In favour of adopting the technology	264 (55)
Against the adoption of the technology	215 (45)

Others: Diseases of the blood and blood-forming organs and certain disorders involving the immune mechanism (5%); factors influencing health status and contact with health services (4%); diseases of the digestive system (3%); diseases of the skin and subcutaneous tissue (3%); symptoms, signs and abnormal clinical and laboratory findings, not elsewhere classified (3%); diseases of the eye and adnexa (2%); mental and behavioural disorders (2%); diseases of the genitourinary system (2%); injury, poisoning and certain other consequences of external causes (1%); diseases of the ear and mastoid process (1%); pregnancy, childbirth and the puerperium (1%)

Public consultations were conducted in 400 processes (83% of the total), in which context 196 483 contributions were made. The median number of contributions per public consultation was 80 (IQR 18–389), with a minimum of zero and a maximum of 41 787 contributions in response to a single public consultation. The PC with the highest number of contributions took place in 2019 for the drug nusinersen for spinal muscular atrophy, which had a preliminary and final recommendation favourable to its adoption into the SUS. Stakeholder categories that participated in PC are presented in Table 2. Most contributions were made by patients and their relatives or representatives (*n* = 99 082; 50%). The characteristics of individual participants are presented in Table 3. More frequently, they were females (122 895; 67%), white individuals (129 165; 71%) and individuals between the ages of 25 and 59 years (145 364; 80%).

**Table 2:** Participation of stakeholder categories in public consultations of the technology incorporation processes into the SUS

Stakeholder	*N* (%)
Patients and families	99 082 (50)
Health institutions and professionals	47 401 (24)
Private companies	1 214 (1)
Public health departments	578 (< 1)
Others	48 326 (25)

**Table 3:** Characteristics of individual participants in public consultations of the technology incorporation processes into the SUS

Characteristics	*N* (%)
*Gender*	
Female	122 895 (67)
Male	59 585 (33)
*Colour or ethnicity*	
White	129 165 (71)
Brown	41 015 (22)
Black	7 888 (4)
Yellow	4 097 (2)
Indigenous	313 (< 1)
*Age group*	
Under 18 years	2 207 (1)
Between 18 and 24 years	16 782 (9)
Between 25 and 39 years	78 676 (43)
Between 40 and 59 years	66 688 (37)
Over 60 years old	17 393 (10)

**Fig. 2:**
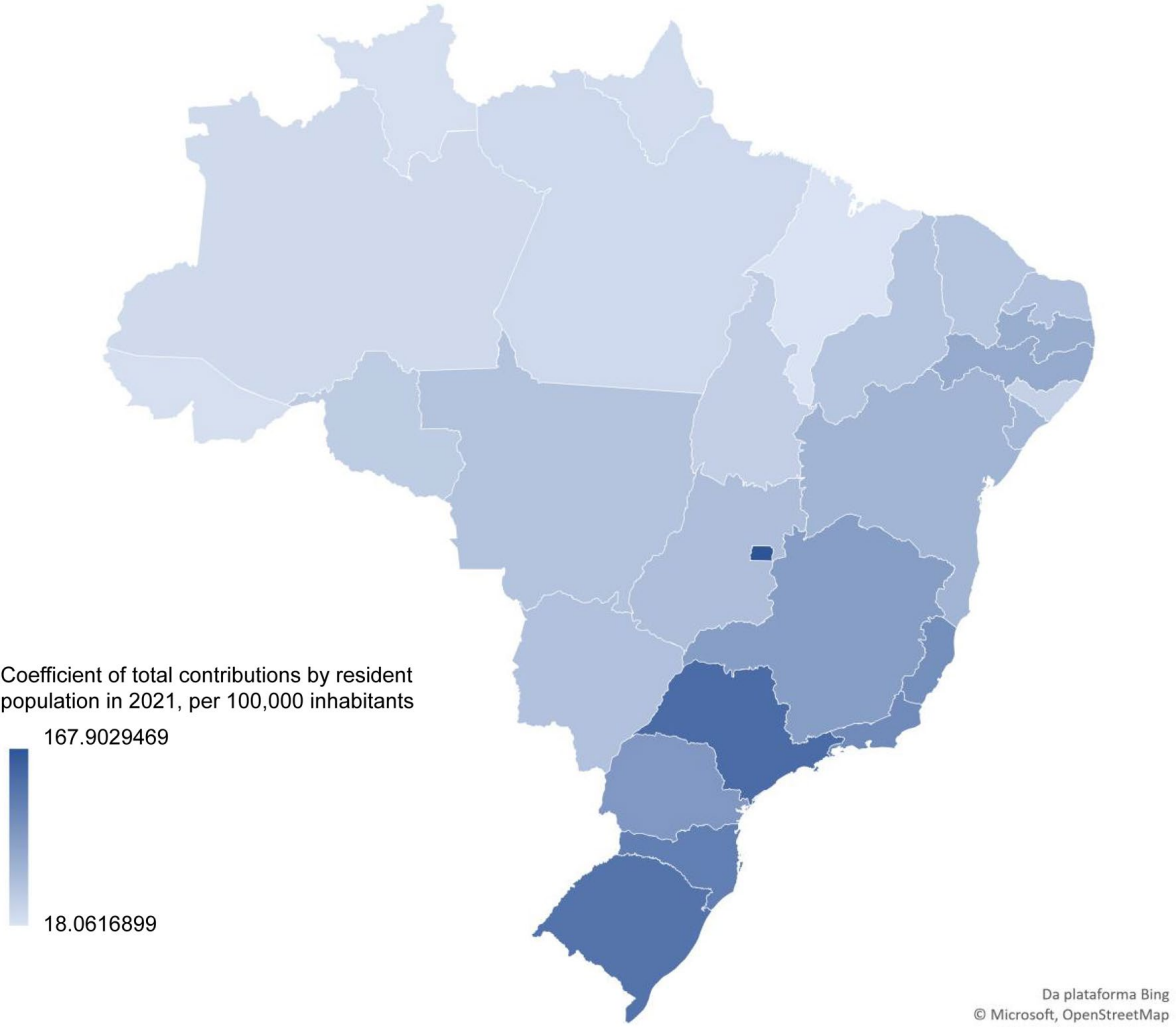
Geographical distribution of participants in public consultations on technology incorporation processes in Brazil

Participants in public consultations did not have a homogeneous geographic origin, as shown in Fig. 2, which reports the coefficient of participation by the resident population of each state in the country. The map highlights the participation of the Federal District and the south and southeast regions of the country.

**Fig. 3:**
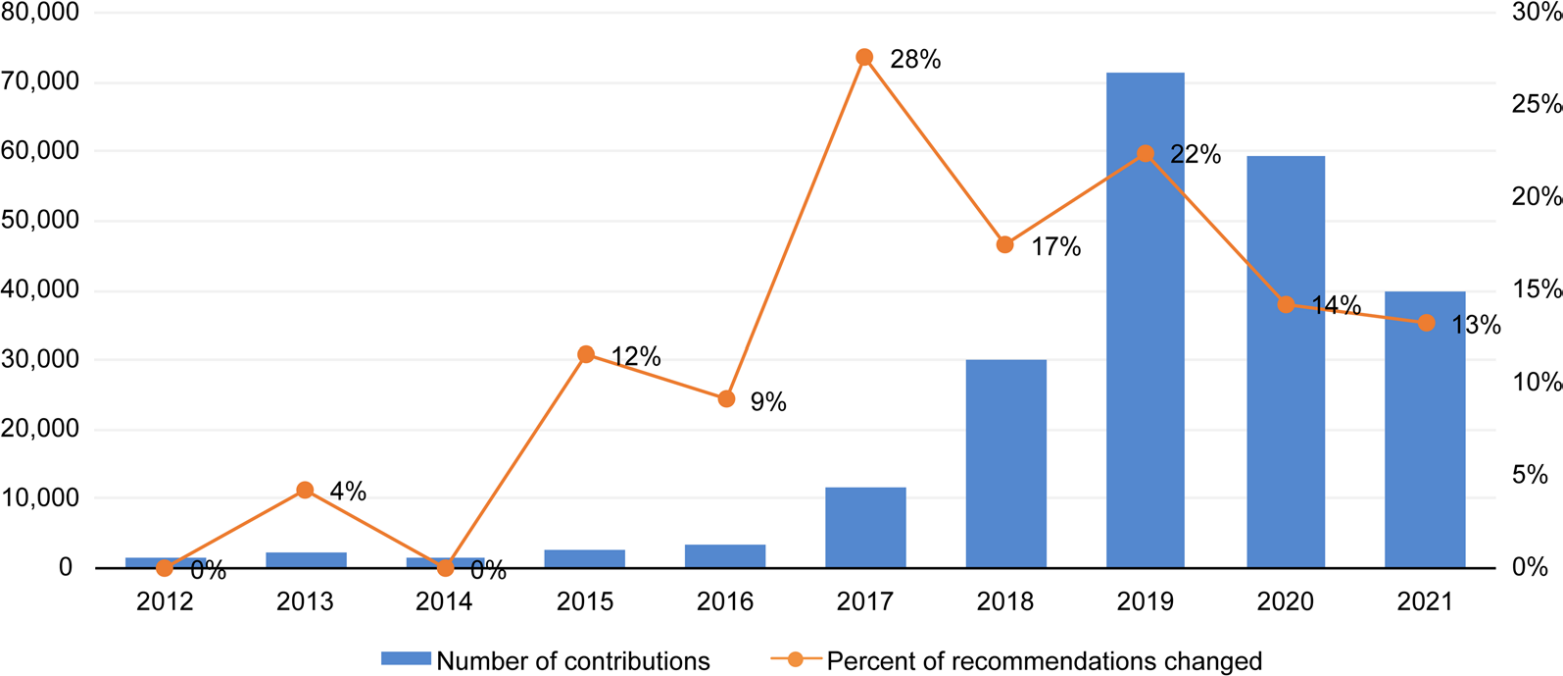
Number of participations in public consultations and proportion of recommendations changed after public consultation

The number of participations in public consultations increased over the years until 2019, which was followed by a decrease over the following 2 years (Fig. 3). The same pattern was observed in relation to the frequency of changes to recommendations following the public consultation, which increased from 2017 onwards. During the period under analysis (2012–2021), the proportion of recommendations that were changed was 13% (*n* = 53) of the recommendations submitted for public consultation. The majority of the 53 cases of changes in these recommendations exhibited the following characteristics: 83% (*n* = 44) focused on medicines, intended to treat diseases of the nervous system (19%; *n* = 10), endocrine, nutritional or metabolic diseases (19%; *n* = 10), and neoplasms (15%; *n* = 8); 62% (*n* = 33) were demanded by the private sector and 96% (*n* = 51) had a final decision in favour of offering the technology in the SUS. One example of a recommendation that was changed after the PC is the case of the rapid-acting insulin analogues for type 1 diabetes, which resulted in a recommendation favourable to the adoption of the medicines into the SUS.

When analysing the possible factors associated with changes in Conitec’s recommendations, it was observed that increased participation on the part of patients and their families had a significant impact on changing these recommendations: odds ratio 3.87, 95% confidence interval 1.33–13.35, *p* = 0.02. That is, the public consultations that received the highest volume of contributions from patients and their families or representatives were nearly four times more likely to change Conitec’s recommendation than public consultations that received the lowest volume of contributions from this group. The other factors under investigation did not exhibit a significant association with changes in the recommendations, even in cases of a higher volume of contributions. The odds ratio, confidence interval and *p*-value of each exposure variable investigated in the model are presented in Table 4.

**Table 4:** Effect of variables on changing Conitec’s recommendation between 2012 and 2021

Variable	OR	95% Confidence interval	*p*-Value
*Intercept*	0.21	0.03–1.08	0.08
*Type of technology:* medicines	0.70	0.29–1.76	0.43
*Demander*			
Government	0.39	0.09–2.14	0.24
Private sector	0.69	0.17–3.61	0.63
Social	0.44	0.04–3.64	0.45
*Indication*			
Neoplasms	0.81	0.28–2.08	0.67
Infectious and parasitic diseases	0.45	0.07–1.71	0.31
Endocrine, nutritional and metabolic diseases	0.94	0.36–2.29	0.90
Diseases of the musculoskeletal system and connective tissue	0.84	0.26–2.28	0.74
*Number of contributions in public consultation*			
Large volume of contributions: total of participants	1.22	0.33–3.84	0.75
Large volume of contributions: patients	**3.87**	**1.33–13.35**	**0.02**
Large volume of contributions: public health institutions and professionals	1.07	0.44–2.70	0.87
Large volume of contributions: other participants	0.97	0.40–2.30	0.94
Number of contributions: private sector	1.03	0.97–1.07	0.25
Number of contributions: public health managers	0.91	0.77–1.03	0.19

Bold values highlight the results in which the variable showed a statistically significant association with the outcome under analysis

Large volume of contributions refers to the group of public consultations that received a number of contributions equal to or greater than 100 for each specified stakeholder. For private sector and public health managers, there was no public consultation with 100 contributions or greater, and the values were included in the model as a continuous variable

## Discussion

The first 10 years of Conitec’s operation featured growth in the implementation of the requirement of patient and public involvement, as evidenced by the number of public consultations conducted and contributions received over the years. In addition, 13% of recommendations changed after the public consultation, and the significant participation of patients and their families was strongly associated with changes in preliminary recommendations regarding the adoption of technologies into the SUS. Previous investigations, which featured shorter follow-up times or analysed specific subgroups, found the frequency of change in recommendations following a public consultation to 18.8% in the case of medicines up to June 2016 [14], 8% in the case of medicines, products and procedures by 2018 [5], and 19% in the case of rare diseases by June 2019 [15].

Even though the participation mechanism adopted had only an advisory nature, the results indicate that people who are directly affected by the health condition or the technology being evaluated may have a greater influence on decisions regarding the adoption of technologies into the SUS than other stakeholders.

On the other hand, the study identified significant disparities in participation rates, particularly in terms of colour/ethnicity and geographic location. Specifically, 71% of contributions were made by self-declared white individuals, while only 20% came from people residing in the north and northeast regions. These findings are likely reflective of broader socioeconomic inequalities in Brazil, where infrastructure (technology and internet) and education are limited for those with unmet health needs, and information is not easily accessible [16, 17].

Silva et al. constructed a timeline of the PPI actions taken in Conitec’s technology incorporation processes until December 2017, which enhances our understanding of the rise in the number of contributions made during the period under analysis [6]. The actions included revising the public consultation forms in 2014, which were developed by Conitec’s PPI team, with separate forms for contributions related to experience or opinions and for those related to technical and scientific points; an increase in the availability of reports in a summarized format using simplified language that targeted lay public in 2015; and publishing a guide titled ‘Understanding the incorporation of health technologies in the SUS: How to get involved’ in 2016 to inform and guide the PPI processes implemented by the institution [6]. It can be inferred that these actions may have contributed to the observed increase in engagement, particularly between 2015 and 2019. In 2019, the public consultation with the highest volume of contributions took place. The drug nusinersen for spinal muscular atrophy, a rare disease, received 41 787 contributions, which represents 59% of 2019 contributions and 21% of all PC contributions.

However, participation rates declined sharply in the following years, 2020 and 2021, which can be attributed to two factors. Firstly, the introduction of a government that was less aligned with participatory practices, including the revocation of the National Policy of Social Participation, which had been instituted in 2014, and eliminated all participatory collegiate bodies that had not been created by law [18]. Secondly, the COVID-19 pandemic, which the federal government responded to with anti-science discourses and practices, further eroded confidence in the government’s actions [19, 20]. In December 2020, Brazil had the second highest rate of the coronavirus worldwide [20], having accumulated a total of more than 34 million confirmed cases and 688 000 deaths by November 2022 [21]. As of December 2022, there remain no national guidelines for outpatient care to people with COVID-19 due to disagreements between Conitec and the MoH [22]. Other health procedures that were unrelated to COVID-19 suffered drastic reductions in 2020 [20]. These political and sanitary factors likely contributed to the decline in social participation observed during 2020 and 2021.

Although the causal relationship cannot be confirmed, some characteristics of this study strengthen the conclusions. First, all processes of medicines, products and health procedures analysed by Conitec during the period of 10 years were included in the regression model, which mitigates concerns related to possible sample bias or a lack of internal validity. Second, the analysis considered the temporal relationship among the events and an objective measurement of the outcome (recommendation changes) preceded by the exposure variable (public consultations). Third, the effect exhibited a large magnitude (OR 3.87) and the positive and significant results concerning the association exhibited high consistency, with a confidence interval that assumes the possibility of even greater effects of the association (95% 1.33–13.35). Finally, the analysis was characterized by specificity in terms of the subgroup in which the effect was observed (exclusively for the subgroup of patients and family members), the dose–response relationship regarding the division of groups into those with higher and lower volumes of participation, and the plausibility of the hypothesis that people who are directly affected by the decision have a greater influence on the decision.

Our study has an important limitation that must be acknowledged: we did not analyse the content of the PC. While we did consider the volume of participation, it is possible that the information presented by participants in their contributions had a greater impact on changing the recommendations than we were able to capture. Furthermore, although the committee members receive the original PC submissions, the content undergoes a summarization and analysis process by a team of specialists responsible for the report. Thus, it is necessary to analyse both the original content and the summary that is made available in public reports to fully understand the impact of PC contributions on the adoption of health technologies into the SUS. Additionally, it is important to note that the influence of PPI and PC contributions on the adoption of health technologies can take place in multiple ways beyond changing recommendation, which should be explored in future investigations.

To enrich our understanding of this subject, there are other aspects that we did not examine in our study but could be valuable to consider. For instance, we could categorize indications into rare and non-rare diseases and examine how decisions are made based on the cost of the technologies in question. The volume of contributions sent by patients in response to public consultations may reflect the prevalence of the health condition in question and, therefore, a differentiation based on the rarity of the disease may be necessary. On the other hand, greater engagement could also be motivated by the adoption of technologies that families are unable to acquire directly for economic reasons or may indicate the increased presence of rare disease organizations, frequently financed by pharmaceutical companies.

Impact evaluations of PPI strategies remain scarce and have found limited or inconsistent results. The systematic review conducted by Boivin and collaborators to investigate PPI assessment instruments indicated a greater focus on the impacts perceived and self-reported by the participants, with most of such assessments being focused on contexts and processes, and fewer assessments emphasizing impacts that are observable by external evaluators [23]. In health decisions informed by HTA, PPI impact assessments have been identified with the result of providing subsidies for HTA studies, such as willingness-to-pay measures or discrete choice questionnaires [12, 24]. In the systematic review of international HTA public engagement experiences conducted by Gagnon et al., the authors concluded that there is a paucity of strong evidence regarding the impact of PPI initiatives, especially in the long term [24]. The results presented in this paper therefore represent an important contribution to our knowledge concerning the influence of PPI on health decisions and can be of interest for other public health systems that have technology management processes mediated by PPI strategies.

Other PPI mechanisms that became available in the process of incorporating technologies into the SUS include (i) demanders on the adoption of technologies and (ii) representatives of the ‘public’ in Conitec committees [5, 7]. It was observed that the presence of ‘social representatives’ in the position of demander remains incipient, representing only 4% (*n* = 17) of all requests analysed during the period from 2012 to 2021. Conitec’s receipt of a request for incorporation depends on the presentation of studies that exhibit a high degree of technical complexity in the form of systematic reviews and analyses of cost-effectiveness and budgetary impacts, which may represent an impediment to this group of stakeholders to access this process.

The representative of the ‘public’ in the Conitec committees holds a fixed appointment regardless of the topic under analysis, and participates directly in the deliberative process involved in the task of evaluating each demand; this representative also has the right to vote. The nomination of this representative is freely chosen by the National Health Council, which represents users, health professionals and the private sector. That is, the person appointed to represent the ‘public’ may have diffuse interests and may not be directly related to each demand. Thus, it is evident that the main mechanism for patients’ social participation is public consultations, which lead to high levels of engagement and allow patients to make themselves heard and to have important impacts on the decision at hand, although from a passive type of PPI.

However, the question of whether the interests presented by patients reflect (exclusively) their own interests or whether patients can serve as spokespersons for other actors remains up for debate. The category of patients and family members referenced in this study grouped the contributions sent both individually and collectively through patient groups and associations. The volume of contributions sent separately by patient associations during the period under analysis corresponds to only 1.4% of the category of patients and family members or 0.7% of total contributions, and it was not possible to determine whether the content of contributions from associations and from individuals matched.

Patients’ associations aim to take actions both in direct support of patients and their families by providing educational, health and emotional support, as well as to articulate public policies to promote access to treatments and other support services, and in this context, strong appeals have been made via social media in recent years [25, 26]. These associations are financed largely by the pharmaceutical industry and tend to reflect the positions of their sponsors [27, 28]. Therefore, it is necessary to investigate not only the influence of the volume of participation in this context, but also the way the contributions of patients represent their interests and needs and the way in which the content thus presented impacts the corresponding decisions.

Despite their frequent use as a PPI mechanism, public consultations are limited by their nature as a one-way form of communication between the public and decision-makers. In accordance with the vision of patient-centred health, PPI practices are encouraged to focus more on dialogue and to emphasize the involvement of all stakeholders at all stages of the decision-making process, rather than merely at the end of the process [29].

Although the rationale for PPI is becoming increasingly consolidated in the scientific literature, its operationalization by institutions that conduct HTA has continued to be challenging [9]. Ivani and Dutilh-Novaes proposed a three-tiered model that can facilitate the analysis of the conditions under which public engagement would be successful in the context of epistemic exchange [29]. According to these authors, the following factors should be considered: (1) the attention or exposure of the actors, in this case, the public institution and society; (2) mutual trust among actors and (3) the form and content of the information included in the epistemic exchange [29].

If a decision regarding the allocation of resources or a recommendation for the adoption of technologies are to be considered fair and reasonable, it is necessary not only to consider the methods that are used and the information that is analysed but also the stakeholders who are included in the process [9]. However, Street et al. (2020) defended clear distinctions among stakeholders because these stakeholders represent different roles and interests, especially with regard to health decisions informed by HTA [8]. In this work, we also found that different groups not only organize themselves and participate in different degrees but also have different influences on the corresponding decisions.

## Conclusions

This work presents the association of patient and public involvement on decisions related to the adoption of technologies into the public health system in Brazil. It was observed that increased engagement of patients and their families and caregivers in public consultations was associated with a nearly four-fold increase in the probability of changing the preliminary recommendation of Conitec during its first 10 years of operation. Despite the increasing interest in the actions taken to promote PPI, the importance of maintaining and improving available spaces and developing more dialogic mechanisms that are less vulnerable to specific political contexts and aimed at establishing a more equitable and accountable health system is worth highlighting.

## Supplementary Information

**Additional file 1. **Complete database with information on demands analyzed by Conitec and characteristics of participants in public consultations.

## Data Availability

All data generated or analysed during this study are included in this published article.

## Abbreviations

MoH: Ministry of Health
REBRATS: Brazilian Network for the Evaluation of Health Technology
PPI: Patient and Public Involvement
PC: Public consultations
Conitec: National Committee for Health Technology Incorporation in the Unified Health System
SUS: Unified Health System
HTA: Health Technology Assessment
CFM: Federal Council of Medicine
AMB: Brazilian Medical Association
IQR: Interquartile range
OR: Odds ratio
CI: Confidence interval
COVID-19: Coronavirus disease

## References

[1] Paim J, Travassos C, Almeida C, Bahia L, MacInko J (2011). The Brazilian health system: history, advances, and challenges. Lancet.

[2] Yuba TY, Novaes HMD, de Soárez PC (2018). Challenges to decision-making processes in the national HTA agency in Brazil: operational procedures, evidence use and recommendations. Health Res Policy Syst.

[3] Lima SGG, Brito C, Andrade CJC (2019). Health technology assessment in Brazil – an international perspective. Cien Saude Colet.

[4] Brasil. Presidência da República. Decreto n^o^ 11.161, de 4 de agosto de 2022. Brasília, DF.; 2022.

[5] Lopes ACF de, Novaes HMD, de Soárez PC. Patient and public involvement in health technology decision-making processes in Brazil. Rev Saude Publica. 2020;54(136):1–10. 10.11606/s1518-8787.2020054002453 PMC7702417 33331420

[6] Silva AS, de Sousa MSA, da Silva EV, Galato D (2019). Social participation in the health technology incorporation process into Unified Health System. Rev Saude Publica.

[7] Silva AS, Facey K, Bryan S, Galato D (2022). A framework for action to improve patient and public involvement in health technology assessment. Int J Technol Assess Health Care.

[8] Street J, Stafinski T, Lopes E, Menon D (2020). Defining the role of the public in Health Technology Assessment (HTA) and HTA-informed decision-making processes. Int J Technol Assess Health Care.

[9] Wale JL, Thomas S, Hamerlijnck D, Hollander R (2021). Patients and public are important stakeholders in health technology assessment but the level of involvement is low—a call to action. Res Involv Engagem.

[10] Modigh A, Sampaio F, Moberg L, Fredriksson M (2021). The impact of patient and public involvement in health research versus healthcare: a scoping review of reviews. Health Policy.

[11] Daykin N, Evans D, Petsoulas C, Sayers A (2007). Evaluating the impact of patient and public involvement initiatives on UK health services: a systematic review. Evidence & Policy.

[12] Gagnon MP, Desmartis M, Lepage-Savary D, Gagnon J, St-Pierre M, Rhainds M (2011). Introducing patients’ and the public’s perspectives to health technology assessment: a systematic review of international experiences. Int J Technol Assess Health Care.

[13] Abelson J, Wagner F, DeJean D, Boesveld S, Gauvin FP, Bean S (2016). Public and patient involvement in health technology assessment: a framework for action. Int J Technol Assess Health Care.

[14] Caetano R, da Silva RM, Pedro ÉM, de Oliveira IAG, Biz AN, Santana P (2017). Incorporation of new medicines by the National Commission for Incorporation of Technologies, 2012 to June 2016. Cien Saude Colet.

[15] Biglia LV, Mendes SJ, de Lima TM, Aguiar PM (2021). Incorporation of drugs for rare diseases in Brazil: Is it possible to have full access to these patients?. Cien Saude Colet.

[16] Szwarcwald CL, Souza Júnior PRBD, Marques AP (2016). Inequalities in healthy life expectancy by Brazilian geographic regions: findings from the National Health Survey. Int J Equity Health..

[17] Coube M, Nikoloski Z, Mrejen M, Mossialos E (2023). Inequalities in unmet need for health care services and medications in Brazil: a decomposition analysis. Lancet Reg Health Am..

[18] Brasil. Presidência da República. Decreto n° 9.759, de 11 de abril de 2019. Extingue e estabelece diretrizes, regras e limitações para colegiados da administração pública federal. Brasília, DF: Diário Oficial da União; 2019.

[19] Hallal PC, Victora CG (2021). Overcoming Brazil’s monumental COVID-19 failure: an urgent call to action. Nat Med.

[20] Bigoni A, Malik AM, Tasca R, Carrera MBM, Schiesari LMC, Gambardella DD (2022). Brazil’s health system functionality amidst of the COVID-19 pandemic: an analysis of resilience. Lancet Reg Health Am..

[21] World Health Organization. WHO COVID-19 Dashboard [Internet]. 2020 https://covid19.who.int/. Accessed 6 Nov 2022.

[22] Correia LCL, Sette C, Santos M, Magliano CAS, Toscas FS (2022). Brazil’s COVID-19 guidelines: political hijack of public health. Lancet.

[23] Boivin A, Espérance AL, Gauvin F, Dumez V, Cm ACM, Lehoux P (2018). Patient and public engagement in research and health system decision making: a systematic review of evaluation tools. Health Expect.

[24] Gagnon MP, Tantchou Dipankui M, Poder TG, Payne-Gagnon J, Mbemba G, Beretta V (2021). Patient and public involvement in health technology assessment: Update of a systematic review of international experiences. Int J Technol Assess Health Care.

[25] Frossard VC, Dias MCM (2016). The impact of internet on patients interaction: new scenarios in health. Interface.

[26] de Lima MA, Gilbert ACB, Horovitz DDG (2018). Treatment networks and associations of patients with rare diseases. Cien Saude Colet.

[27] Fabbri A, Parker L, Colombo C, Mosconi P, Barbara G, Frattaruolo MP (2020). Industry funding of patient and health consumer organisations: systematic review with meta-analysis. The BMJ.

[28] Khabsa J, Semaan A, El-Harakeh A, Khamis AM, Obeid S, Noureldine HA (2020). Financial relationships between patient and consumer representatives and the health industry: a systematic review. Health Expect.

[29] Ivani S, Dutilh NC (2022). Public engagement and argumentation in science. Eur J Philos Sci.

